# A case report of ovarian granulosa cell tumor in patient with polycystic ovarian syndrome

**DOI:** 10.1097/MD.0000000000028261

**Published:** 2021-12-17

**Authors:** Yun S. Kim, Ji H. Lee

**Affiliations:** a Department of Obstetrics and Gynecology, Soonchunhyang University College of Medicine, Soonchunhyang University Cheonan Hospital, Cheonan, Korea; b Department of Pathology, Soonchunhyang University College of Medicine, Soonchunhyang University Cheonan Hospital, Cheonan, Korea.

**Keywords:** adult type granulosa cell tumor, amenorrhea, polycystic ovarian syndrome

## Abstract

**Rationale:**

Granulosa cell tumors (GCTs) account for less than 2% of all ovarian malignancies and are the second most common ovarian sex cord stromal tumors after fibroma/thecomas.

GCTs occur most frequently in postmenopausal women with a peak age of 50 to 55, are usually diagnosed in their early stages, and have a good prognosis. GCTs usually present with features of hyperestrogenism, with an average size is 10 to 15 cm.

**Patient concerns:**

A 31-year-old nulligravida diagnosed with polycystic ovarian syndrome (PCOS) 10 years prior, had a 20-mm mass in her right ovary found on ultrasonography 2 years ago. She had been taking dienogest 2 mg for 2 years for a misdiagnosed endometrioma, but over a 2-year course, the mass increased to 50 mm.

**Diagnoses:**

An ultrasound scan revealed a 47 × 37-mm round solid mass in the right ovary with a spongiform appearance and little vascularity. The pathologic findings showed an adult-type granulosa cell tumor with necrosis and hemorrhage. The tissue stained positive for inhibin-α, Wilms’ tumor-1, CD56, and negative for cytokeratin 7.

**Interventions:**

We finally performed right salpingo-oophorectomy, endometrial biopsy, peritoneal biopsy, and partial omentectomy. The pathological findings were adult-type granulosa cell tumor. The International Federation of Gynecology and Obstetrics staging was IA. The patient did not require additional treatment.

**Outcomes:**

Surprisingly, her normal menstruation returned 2 weeks after the operation, and she had a normal pregnancy and parturition. The patient had been followed-up regularly for 3 years following the surgery. The patient has not experienced any complications and has remained disease-free.

**Lessons:**

GCTs should be considered in the differential diagnosis if a female patient with PCOS and amenorrhea shows a unilateral small solid mass. They are extremely rare malignant ovarian tumors that must be differentiated from other benign ovarian tumors, especially endometriomas and dermoid cysts. It was difficult for us to suspect a granulosa cell tumor because the patient already had PCOS symptoms such as mild hirsutism and amenorrhea. This case highlights the importance of physicians being aware of and suspicious for GCTs in similar cases, along with knowing their characteristics in considering possible differential diagnoses.

## 1 Introduction

Malignant neoplasms originating from granulosa cells of the ovarian follicles account for approximately 10% of all sex cord stromal tumors of the ovary. Most GCTs were adult type (95%), and 5% were juvenile type. The most common age in postmenopausal women peaks at the age of 50 to 55 years.^[1]^ Most are confined to the ovary (stage I) and rarely metastasize to the lung or liver. Lymph node metastases are rare, with more than 95% being unilateral and confined to the ovaries. They are usually encapsulated with smooth lobulated surfaces, tan or yellow (depending on the degree of luteinization and lipid content), soft to firm (depending on the amount of fibromatous component), usually solid and cystic with straw-colored or mucoid fluid and can have areas of necrosis and hemorrhage. More luteinized tumors are more yellow/orange.^[2]^ This paper reports an unusual case of adult type granulosa cell tumor (AGCT) with manifestations that include amenorrhea, mild hirsutism, dienogest resistance, normal testosterone, normal estrogen, and high anti-Müllerian hormone (AMH) levels.

## 2 Case presentation

**Figure 1:**
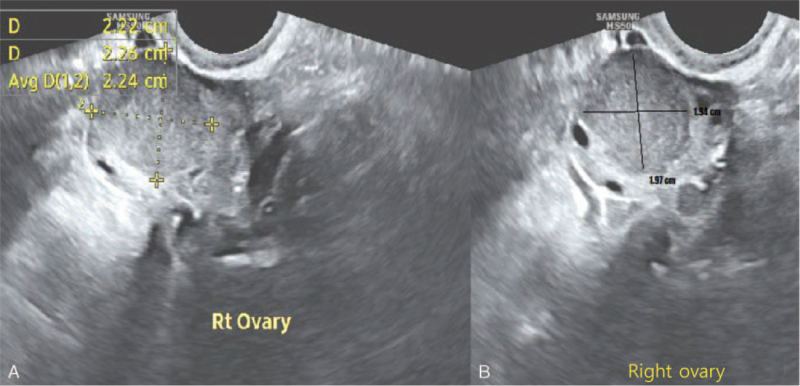
Ultrasonographic findings taken at local clinic 2 yrs ago. (A) A 20 × 37-mm sized right ovarian mass was incidentally on an ultrasound taken at local clinic 2 yrs ago. (B) The mass was thought to be ovarian endometrioma.

**Figure 2:**
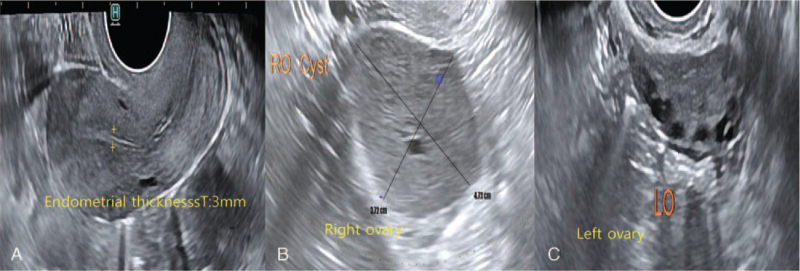
An ultrasound scan at admission. (A) The endometrial thickness was 3 mm. (B) An ultrasound scan revealed a 47 × 37-mm-sized round solid mass on the right ovary with a spongiform appearance and little vascularity. (C) The left ovary showed a PCO pattern.

**Figure 3:**
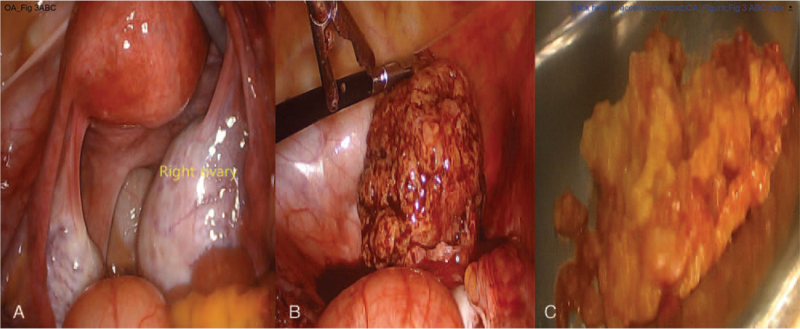
Initial laparoscopic findings. (A) A right ovarian mass was seen. The left ovary appeared normal. (B) The mass was not ruptured during procedure. We placed it in a lap bag. (C) The mass removed with a spoon was yellow and brittle.

**Figure 4:**
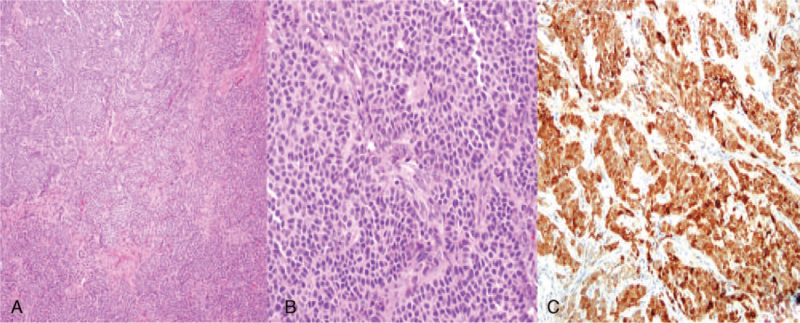
Microscopic findings. (A) The tumor showed variable architectural patterns consisting of solid nests, trabeculae, and microfollicular pattern in the fibromatous stroma (H&E, ×100). (B) Tumor cells had uniform, pale, round-to-oval nuclei with an irregular nuclear membrane, nuclear grooves, and scant cytoplasm (H&E, ×400). (C) Tumor cells were positive for inhibin-A (×200).

**Figure 5:**
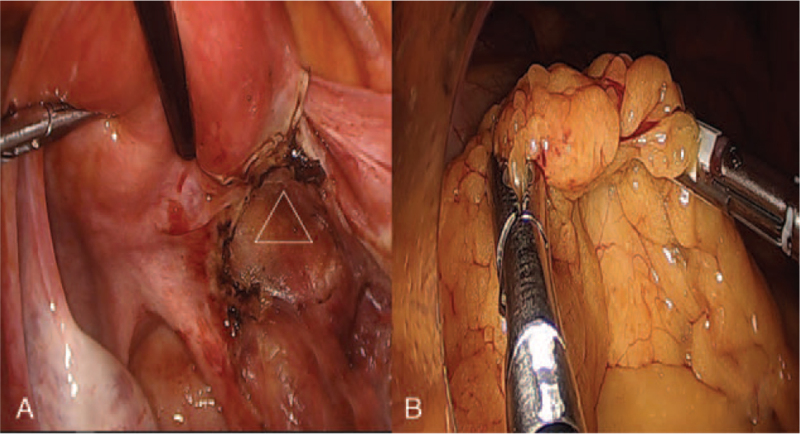
Final laparoscopic findings 2 wks following initial operation. (A) We performed pelviscopic right salpingo-oophorectomy (Δ: resected area). (B) We also performed partial omentectomy.

A 31-year-old Asian nulliparous woman with a right ovarian mass that had been increasing in size. She had no medical or surgical history, was 160 cm tall, and weighed 60 kg at the time of her visit. She had been diagnosed with polycystic ovarian syndrome (PCOS) 10 years previously and had been administered progesterone injections every 3 months because of irregular menstrual periods. She was incidentally diagnosed with a 20-mm sized right ovarian mass on ultrasound performed at a local clinic 2 years ago. This was thought to be an ovarian endometrioma, so she took dienogest for 2 years (Fig. 1A, B). However, over the 2-year period, the mass increased to 50 mm. She stopped taking dienogest and came to the hospital 1 week ago. An ultrasound scan revealed a 47 × 37-mm round solid mass in the right ovary, with a spongiform appearance and little vascularity. The endometrial thickness was 3 mm, and the left ovary showed a PCO-type pattern (Fig. 2A, B, C). The tumor marker levels, including alpha-fetoprotein, CA 19–9, CA 125, and carcinoembryonic antigen, were not elevated. Hormonal analyses were as follows: Estradiol levels: 43.9 pg/mL (reference range 27-433 pg/mL); luteinizing hormone (LH): 25.2 mIU/mL, follicle stimulating hormone (FSH): 5.11 mIU/mL, testosterone: 0.26 ng/mL (0.084-0.481 ng/mL). The AMH level was 23.60 ng/mL, which is the 90^th^ to 100^th^ percentile of the same age group. Chest radiography did not reveal any remarkable findings. Therefore, an initial diagnosis of benign right ovarian endometrioma or dermoid cyst was made. The patient underwent laparoscopic right ovarian cystectomy, and the mass did not rupture during the procedure. The mass was yellow and brittle. The mass was placed in a lap bag and removed with a spoon (Fig. 3A, B, C). The ovarian tumor findings on frozen section analysis indicated that AGCT could be a possibility. Accordingly, the patient's parent decided to choose the next surgical procedure after obtaining a final pathological diagnosis. Microscopic examination of the right ovarian mass revealed diffuse, solid, cordlike, and trabecular admixed architectural patterns (Fig. 4A). We also noted Call-Exner bodies. The tumor cells had fine chromatin and round-to-oval nuclei with a single small nucleolus. Some nuclei, known as “coffee-bean” nuclei, showed pale, round longitudinal grooves (Fig. 4B). Mitotic figures are rare. Fibromatous stroma was also observed. Tumors stained positive for inhibin-α, Wilms’ tumor (WT)-1, and CD56, and negative for CK7 (Fig. 4C). Thus, the findings supported the final diagnosis of AGCT in the right ovary. We performed pelviscopic right salpingo-oophorectomy, endometrial biopsy, peritoneal biopsy, and partial omentectomy 2 weeks after the initial operation (Fig. 5A, B). Microscopic examination revealed no residual AGCT cells. An endometrial biopsy revealed no hyperplasia. Additionally, the tumor was classified as Federation of Gynecology and Obstetrics stage IA. The patient did not require additional treatment. Surprisingly, her normal menstruation returned 2 weeks after the operation, and she had a normal pregnancy and parturition. The patient had been followed-up regularly for 3 years following the surgery. The patient has not experienced any complications and has remained disease-free.

## 3 Discussions

GCTs are derived from the granulosa cell layer of the ovarian follicles. Normally, these cells proliferate in response to rising circulating gonadotropins and decline in response to circulating testosterone. The granulosa and theca cells are part of the ovarian follicle and form the cellular stroma surrounding the developing oocyte. They normally produce estradiol. Excess estradiol, when sensed by the hypothalamus and anterior pituitary, decreases the production of gonadotropin-releasing hormone, FSH, and LH.^[3]^ Stimulation of granulosa cells by activin increases FSH binding and FSH-induced aromatization in follicles. This also enhances the action of LH on theca cells of the ovary to enhance androgen synthesis. Granulosa cells also produce AMH and inhibins A and B. Inhibin B is produced as a result of FSH stimulation and provides feedback to the pituitary to decrease FSH production. Excess estradiol produced by the tumor often results in abnormal uterine bleeding, postmenopausal bleeding, and abdominal pain.^[4]^ In women who still possess a uterus, excess estradiol from granulosa cell tumor (GCT) leads to an increase in endometrial hyperplasia and an increased risk of breast cancer.^[5]^ GCTs are subdivided into AGCT and juvenile GCT (JGCT), indicating not only the typical age of the patient and the differentiating histologic features, but also the differing natural history. However, both forms of GCTs may occur in any age group. On reviewing the literature, we found that two-thirds of all AGCT cases were present in postmenopausal women, with a peak incidence between 50 and 55 years, whereas 1% of AGCT cases were observed in prepubescent girls. JGCTs are mainly present in prepubescent girls and women aged < 30 years, with more than half of the cases in patients aged < 10 years.^[6]^ On ultrasound examination, most GCTs are large multilocular solid masses with a large number of locules or solid tumors with heterogeneous echogenicity of the solid tissue. Hemorrhagic components are common, and increased vascularity has been demonstrated by color/power Doppler ultrasound examination.^[7]^ The hyperestrogenic state that is created by the tumor often causes endometrial pathology with bleeding problems as a typically associated symptom. Radiological findings include irregular, thick walls and septa, papillary projections, and solid and echogenic foci.^[8,9]^ Histologically, GCTs are typically characterized by Call-Exner bodies and coffee bean nuclei. On pathology, the solid and cystic masses had GCTs with macrofollicular and microfollicular patterns. The solid masses with a spongiform appearance had prominent hemorrhagic necrosis and diffuse proliferation of granulosa cells with trabecular and microfollicular patterns. The classic features of AGCT on histological examination include Call-Exner bodies and “Coffee-bean” nuclei, along with a low mitotic rate. Call-Exner bodies are gland-like structures that appear similar to those of ovarian follicles. “Coffee-bean” nuclei are pale, round, and longitudinally grooved. In contrast, JGCTs have only a few typical findings. JGCTs have fewer Call-Exner bodies, and gland-like structures resembling ovarian follicles are irregular in size and shape.^[10]^ In addition, JGCTs have immature nuclei with atypia and increased mitotic activity. Immunohistochemistry revealed that granulosa cells displayed stable expression of inhibin-α, WT-1, and CD56. Based on the combination of inhibin-α, WT-1, and positive CD56, pathologists can always be confident in the diagnosis of GCT.^[11]^ In the above case, the epithelial cells of the ovarian tumors were also positive for inhibin-α, WT-1, and CD56. Surgical staging is the most important indicator of prognosis, recurrence, and treatment.^[12]^ Surgery is the cornerstone treatment for both primary and relapsed tumors, whereas chemotherapy is used only for advanced or irresectable tumors. Tumor stage is the only factor consistently associated with prognosis. However, one-third of patients typically relapse within 4 to 7 years following diagnosis, leading to death in 50% of these patients. AMH and inhibin B are currently the most accurate circulating biomarkers that correlate with disease status. Small series have reported low recurrence rates in Stage I (5.4%), rising to 21.1% and 40% in stages III and IV.^[13,14]^ Adjuvant chemotherapy is usually offered to patients who are diagnosed with stage IC with excrescences on the surface of the ovary and at more advanced stages. Unilateral salpingo-oophorectomy is the primary treatment for early stage GCT tumors.^[15]^ Stage I (confined to the ovary) is the most common presentation, and surgery is mostly curative. As with other tumors of mesenchymal origin, lymphadenectomy has not been considered to play a role in the management of these patients.^[16]^ In a recent analysis, completely surgically staged patients fared significantly better. Adjuvant therapies and traditional chemotherapies are administered in cases of high stage disease (stage IC or higher).^[17]^ Since overproduction of estradiol from the tumor is a feature of GCTs, uterine pathology is common. The process of addressing atypical hyperplasia or endometrial carcinomas subsequently leads to total hysterectomy and resection of a unilateral adnexal tumor. Endocrine therapies have been introduced as treatments principally as second- or third-line therapies following the failure of 1 or more lines of chemotherapy. Endocrine therapies were initially introduced with the rationale of interrupting hormone receptor signaling to achieve antitumor effects in a mechanism that is analogous to what is done in the treatment of hormone receptor-positive breast cancer. Initial regimens include tamoxifen and later progestins to modulate estrogen receptor function. Later, regimens added gonadotropin-releasing hormone agonists to suppress ovarian function.^[18,19]^ More recently, aromatase inhibitors have played an increasingly important role in GCT management. GCTs are characterized by late recurrences following resection, sometimes as long as 20 years, but with subsequent increasing aggressiveness. Five- year disease-free survival rates are high (approximately 90%) for early stage disease. However, once a patient has a recurrence, the likelihood of death from the disease increases. Metastases occur mostly locally within the peritoneal cavity, but not infrequently, spreading to the lungs, liver, and spleen, but rarely to the bone and brain. Spread to regional lymph nodes is also rare, but may occur more frequently in higher-stage disease.^[20]^ The purpose of this study was to highlight the difficulty of diagnosing an uncommon small-sized GCT in a 31-year-old PCOS patient. This is worth because, to date, there have been few reports of AGCT associated with PCOS patients. If a woman with PCO develops a small ovarian mass at an atypical age and is resistant to dienogest, GCT should be differentially diagnosed, even if similar symptoms are present.

## Acknowledgments

The authors are grateful to Soonchunhyang University Cheonan Hospital for their assistance and encouragement.

## Author contributions

Conceptualization, data curation, investigation, and writing of the original draft preparation: Kim YS. Writing-review, Pathology: Lee JH.

All authors read and approved the final manuscript.

**Conceptualization:** Yun Sook Kim.

**Data curation:** Yun Sook Kim.

**Investigation:** Yun Sook Kim.

**Writing – original draft:** Yun Sook Kim.

**Writing – review & editing:** Ji Hye Lee.
